# Lobectomy versus lung-sparing resection for congenital pulmonary airway malformation (CPAM): a single-center comparative study

**DOI:** 10.1007/s00383-026-06509-0

**Published:** 2026-06-29

**Authors:** Irene Paraboschi, Ugo Maria Pierucci, Carlotta Ardenghi, Michela Marinaro, Michele Ceresola, Eleonora Durante, Sara Baroni, Salvatore Zirpoli, Michele Ghezzi, Francesca Izzo, Anna Mandelli, Sara Costanzo, Gloria Pelizzo

**Affiliations:** 1 Department of Biomedical and Clinical Science, University of Milano, Milan, Italy; 2 Department of Pediatric Surgery, “Vittore Buzzi” Children’s Hospital, Milan, Italy; 3 Pediatric Radiology Unit, “Vittore Buzzi” Children’s Hospital, Milan, Italy; 4 Pediatric Department, “Vittore Buzzi” Children’s Hospital, Milano, Italy; 5 Anesthesia and Intensive Care Unit, “Vittore Buzzi” Children’s Hospital, Milan, Italy

**Keywords:** Rotator cuff tears, Deltoid muscle, Remaining rotator cuff muscles, Compensatory activation, Real-time tissue elastography

## Abstract

**Aim:**

To compare perioperative and long-term outcomes of lobectomy versus lung-sparing resection in children undergoing surgery for congenital pulmonary airway malformation (CPAM).

**Methods:**

A retrospective single-center study was conducted including children who underwent primary surgical resection for postnatally confirmed CPAM between 2005 and 2024. Patients with other congenital lung lesions, bilateral disease, syndromic conditions, or incomplete data were excluded. Perioperative variables, postoperative outcomes, redo surgery, and chest wall anomalies were compared between lobectomy and lung-sparing resections. Continuous variables were analyzed using the Mann–Whitney U test and categorical variables using Fisher’s exact test.

**Results:**

Thirty-one children were included (22 lobectomies, 9 lung-sparing resections), with a median age at surgery of 8 months. Operative time (207 vs. 149 min, *p* = 0.0425) and anesthesia time (371 vs. 230 min, *p* = 0.0171) were significantly longer for lobectomy. Intraoperative complications occurred in 10% of patients, with no significant difference between groups. Postoperative complications were less frequent after lobectomy (19% vs. 44%), although not statistically significant (*p* = 0.1954). Redo surgery was required exclusively after lung-sparing resections (33% vs. 0%, *p* = 0.0207). Length of hospital stay and NICU/PICU stay were comparable. At a median follow-up of 71 months, chest wall deformities were observed less often after lobectomy (22% vs. 43%, *p* = 0.3554).

**Conclusions:**

Despite longer operative and anesthesia times, lobectomy provided more definitive disease control, with a significantly lower need for redo surgery compared with lung-sparing resection. Lobectomy remains the most reliable surgical option for CPAM in children, while lung-sparing approaches should be reserved for carefully selected cases.

## Introduction

Congenital pulmonary airway malformation (CPAM) represents the most frequent developmental anomaly of the lower respiratory tract, arising from abnormal branching morphogenesis of the fetal airway and leading to cystic or solid lesions of varying size and distribution. With the widespread use of prenatal ultrasonography and fetal magnetic resonance imaging (MRI), the incidence of prenatally diagnosed congenital lung malformations has progressively increased over recent decades [1]. Although asymptomatic at birth in most children, CPAM carries potential long-term risks, including recurrent infection, pneumothorax, respiratory distress, and, in rare cases, malignant transformation [1, 2]. For these reasons, early surgical resection remains the treatment of choice for both symptomatic and radiologically detected lesions. Traditionally, lobectomy has been considered the standard surgical approach for CPAM, ensuring complete excision of the malformation and minimizing the risk of residual disease [3, 4]. Several large pediatric series have demonstrated the safety and efficacy of lobectomy, with low rates of major complications and favorable long-term pulmonary function outcomes [1, 2]. Moreover, compensatory lung growth after lobectomy allows functional recovery of the remaining parenchyma in children, supporting the concept that radical resection at an early age does not compromise future respiratory performance.

In recent years, however, the development of advanced thoracoscopic techniques and improved perioperative management has encouraged some surgeons to explore parenchyma-sparing resections, including anatomical segmentectomy or wedge resection, with the rationale of preserving healthy lung tissue [3, 5, 6]. Advocates of this approach report comparable short-term morbidity and, in selected cases, equivalent postoperative lung function[2, 7].

Nonetheless, lung-sparing surgery remains technically demanding, especially in small children, due to the narrow operative field, anatomical variability, and the risk of incomplete excision [3, 5, 6]. Concerns persist regarding residual or recurrent disease, persistent air leakage, and the need for redo surgery [4, 6].

The aim of this study was to compare the perioperative and postoperative outcomes of lobectomy and lung-sparing resection for CPAM in children, evaluating the balance between operative burden, postoperative complications, and surgical efficacy.

## Materials and methods

A retrospective review was conducted of all pediatric patients who underwent surgical treatment for congenital lung malformations at our Institution between January 2005 and December 2024. During this period, all children referred for surgical management of a congenital pulmonary lesion were initially screened.

**Fig. 1:**
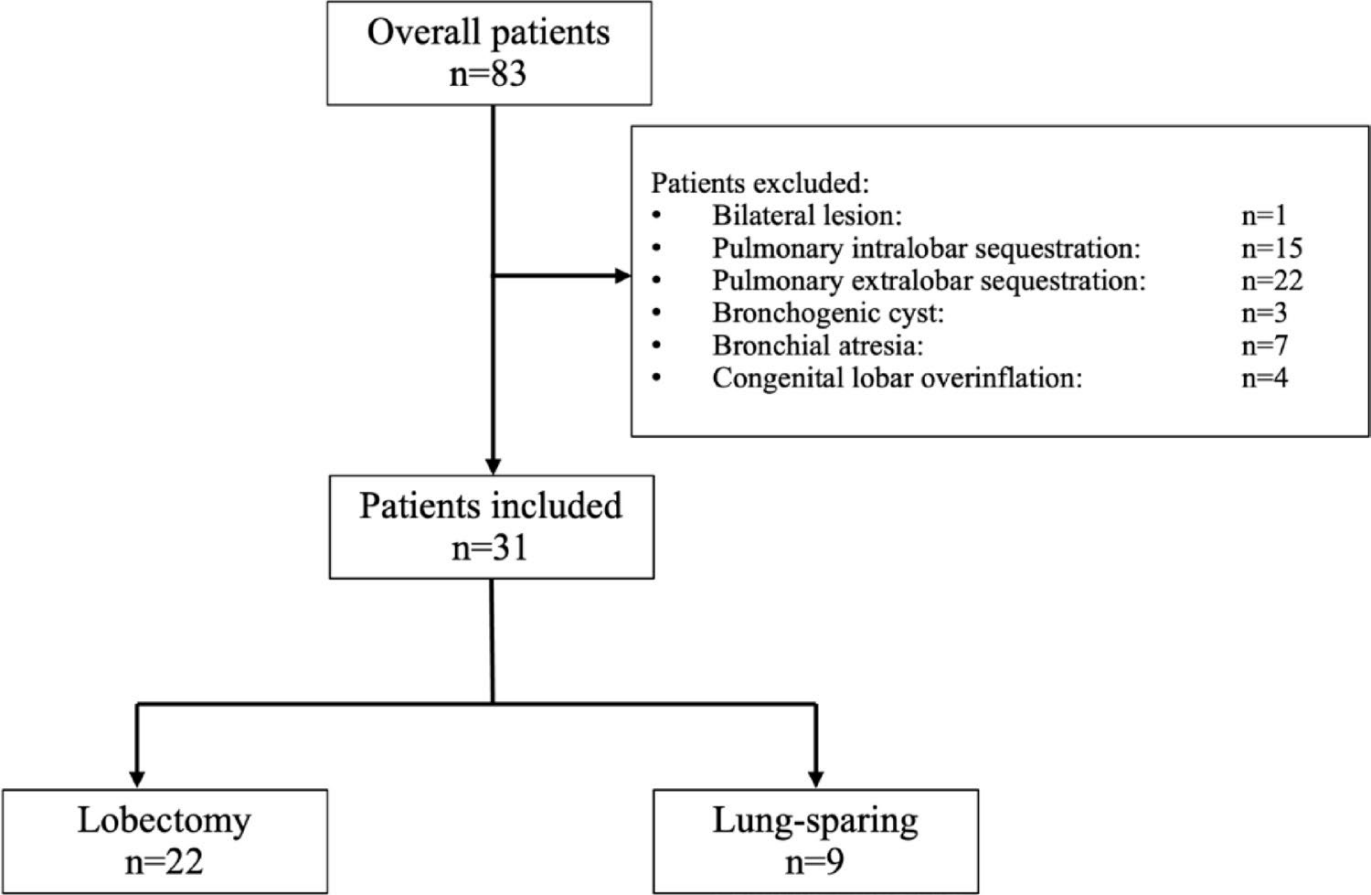
Flowchart of patient selection and study cohort stratification

To obtain a homogeneous and clinically meaningful cohort, a stepwise selection process was applied (Fig. 1). First, only patients with a postnatally confirmed diagnosis of CPAM were considered eligible. Patients with other congenital lung anomalies, including pulmonary sequestration (intralobar or extralobar), bronchogenic cysts, bronchial atresia, congenital lobar overinflation, or hybrid lesions, were excluded.

Second, patients with bilateral pulmonary involvement, associated major thoracic malformations, or syndromic conditions potentially affecting postoperative respiratory outcomes were excluded to limit confounding factors.

Third, only children who underwent primary surgical resection at our Institution were included. Patients referred for redo surgery after an initial operation performed elsewhere were excluded.

Finally, inclusion was restricted to patients with complete and retrievable datasets, including prenatal imaging, operative reports detailing the extent of resection, perioperative variables, and postoperative follow-up data. Patients with missing key variables or insufficient follow-up information were excluded from the final analysis.

After application of these criteria, a total of 31 children constituted the final study population and were included in the comparative analysis between lobectomy (*n* = 22) and lung-sparing resection (*n* = 9).

All procedures were performed under general anesthesia. The choice between lobectomy and lung-sparing resection, as well as the surgical approach (thoracotomy or thoracoscopy), was determined by the operating surgeon’s preference and intraoperative assessment. Two main surgeons were involved in all procedures, both experienced in pediatric thoracic surgery.

Perioperative parameters included operative and anesthesia times, intraoperative complications, need for transfusion, conversion to open surgery, and length of stay in the intensive care unit (NICU/PICU) and hospital. Postoperative outcomes comprised early complications, need for redo surgery, and long-term sequelae such as chest wall deformities. Chest wall anomalies were defined as any clinically or radiologically detectable alteration of thoracic symmetry or spinal alignment observed during follow-up. Specifically, the following conditions were included: thoracic asymmetry or rib cage deformity (including unilateral flattening, protrusion, or asymmetrical chest wall growth), pectus deformities (pectus excavatum or pectus carinatum), and thoracogenic scoliosis, defined as a lateral spinal curvature developing after thoracic surgery and documented on clinical examination and/or radiological assessment. Chest wall anomalies were identified during routine outpatient follow-up based on physical examination and, when clinically indicated, confirmed by chest radiography or thorax MRI. Only deformities considered persistent and clinically relevant at last follow-up were recorded for the purpose of analysis.

Continuous variables were expressed as median and interquartile range (IQR), and categorical variables as counts and percentages. Comparisons between groups (lobectomy vs. lung-sparing) were performed using the Mann–Whitney U test for continuous data and Fisher’s exact test for categorical data. A p-value < 0.05 was considered statistically significant.

## Results

A total of 31 children who underwent surgery for CPAM were included in the study (Table I). The cohort comprised 18 (58%) males and 13 (42%) females. The median age at surgery was 8 (IQR: 2–8) months. A thoracoscopic approach was used in 43% of procedures, while 57% were performed through thoracotomy. Lobectomy was carried out in 22 patients (71%), and lung-sparing resections (segmentectomy or wedge) in 9 patients (29%).

**Table 1:** Baseline demographic, prenatal, perinatal, and surgical characteristics of the study population

Variables	
*Gender (n, %):*	
Female	13 (42%)
Male	18 (58%)
* Side of the lesion (%):*	
Right	17 (55%)
Left	13 (42%)
nd	1 (3%)
*Delivery method:*	
Vaginal birth	19 (61%)
Cesarean section	11 (36%)
nd	1 (3%)
Birth weight *(grams)*	3,110 (2,823 – 3,478) [n=30]
Head circumference *(centimeters)*	33.0 (32.0 – 34.5) [n=15]
Length *(centimeters)*	50.0 (48.0 – 51.0) [n=15]
Gestational age *(gestational weeks)*	39^+1^ (37^+0^ – 40^+2^) [n=29]
Prenatal diagnosis *(n, %)*	27 (90%) [n=30]
CVR max *(n)*	0.860 (0.740 – 1.100) [n=4]
Maternal gestational issues *(%)*	7 (35%) [n=20]
APGAR score 1’ *(n)*	9 (9-9) [n=20]
APGAR score 5’ *(n)*	10 (9–10) [n=20]
Associated anomalies at birth *(%)*	12 (40%) [n=30]
Cardiovascular anomalies *(%)*	7 (24%) [n=29]
Urinary anomalies *(%)*	3 (10%) [n=29]
Central nervous system anomalies *(%)*	2 (7%) [n=29]
Gastrointestinal anomalies *(%)*	2 (7%) [n=29]
Gene/chromosomal anomalies *(%)*	1(3%) [n=30]
*ASA score (n):*	
1	0 (0%)
2	10 (32%)
3	16 (52%)
4	1 (3%)
5	2 (6%)
nd	2 (6%)
Age at surgery *(months)*	8 (2 – 8) [n=31]
*Surgical technique (%):*	
Thoracoscopy	13 (43%)
Thoracotomy	17 (57%)
*Type of surgery (%):*	
Lobectomy	22 (71%)
Lung-sparing	9 (29%)
Operative time *(minutes)*	190.0 (149.0 – 275.0) [n=29]
Anesthesia time *(minutes)*	321.0 (268.3 – 401.8) [n=28]
Intraoperative complications *(%)*	3 (10%) [n=30]
Postoperative complications *(%)*	27 (%) [n=30]
Redo surgery *(%):*	3 (10%) [n=30]
Length of NICU/PICU stays *(days)*	3 (2 – 6) [n=28]
Length of hospital stays *(days)*	10 (7 – 15) [n=30]
Wall chest deformities *(%)*	8 (28%) [n=25]
Follow-up *(months)*	71 (30 – 134) [n=24]

The median operative time was significantly longer for lobectomy compared with lung-sparing resections (207 [176–293] vs. 149 [100–185] minutes, *p* = 0.0425), as was the anesthesia time (371 [294–434] vs. 230 [203–322] minutes, *p* = 0.0171). Intraoperative complications (*n* = 2 intraoperative ventilatory failure; *n* = 1 bleeding) occurred in 10% of patients overall, with no significant difference between the two groups (*p* = 0.5345).

Although not statistically significant, postoperative complications (*n* = 7 persistent pneumothorax, *n* = 1 postoperative bleeding) were less frequent after lobectomy compared with lung-sparing resections (19% vs. 44%, *p* = 0.1954).

The redo surgery rate was significantly higher after lung-sparing resections (33% vs. 0%, *p* = 0.0207). Conversion to open surgery occurred only in thoracoscopic lobectomies (57%). The length of hospital stay (10 [7–15] days) and PICU stay (3 [2–6] days) did not differ significantly between groups.

**Fig. 2:**
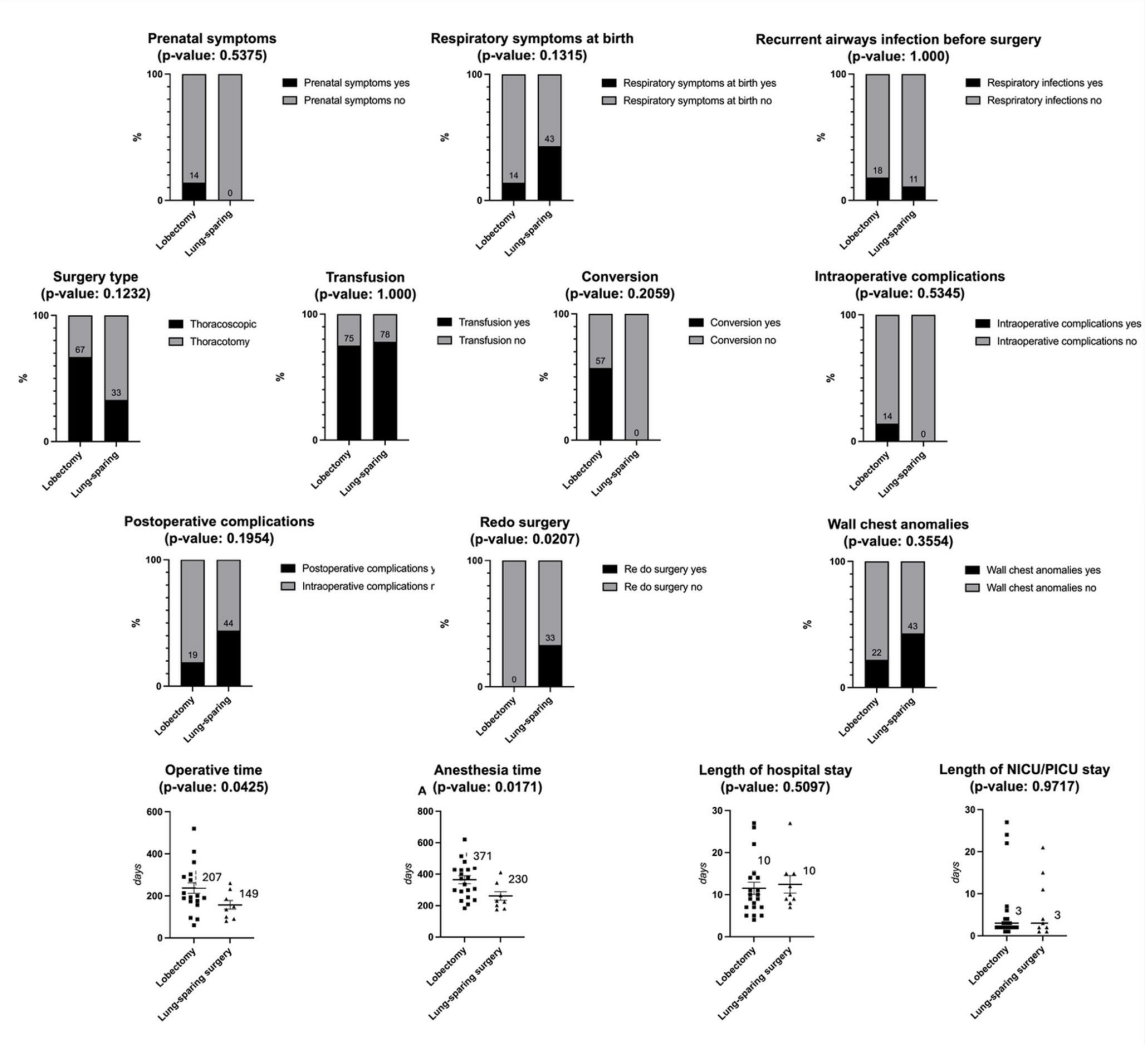
Comparative analysis of clinical presentation, perioperative variables, and outcomes between lobectomy and lung-sparing surgery groups

At a median follow-up of 71 (30–134) months, chest wall deformities (*n* = 2 pectus excavatum; *n* = 2 pectus carinatum; *n* = 2 scoliosis; *n*=1costal anomalies) were observed less often following lobectomy (22% vs. 43%, *p* = 0.3554) (Fig. 2, Table II).

**Table 2:** Comparative analysis of perioperative and postoperative outcomes between patients undergoing lobectomy and lung-sparing surgery

	Lobectomy (n=22)	Lung-sparing (n=9)	p-**V**alue
Age at surgery *(months)*	6 (4 – 9) [n=22]	6 (1 – 8) [n=9]	0.6287
*Surgery type (%):*			
Open	7 (33%) [n=21]	6 (67%) [n=9]	0.1232
Thoracoscopy	14 (67%) [n=21]	3 (33%) [n=9]	
Intraoperative complications *(%)*	3 (14%) [n=21]	0 (0%) [n=9]	0.5345
Transfusion *(%)*	15 (75%) [n=20]	7 (78%) [n=9]	1.000
Conversion *(%)*	8 (57%) [n=14]	0 (0%) [n=3]	0.2059
Postoperative complications *(%)*	4 (19%) [n=21]	4 (44%) [n=9]	0.1954
Operative time *(minutes)*	207 (176 – 293) [n=20]	149 (100 – 185) [n=9]	0.0425
Anesthesia time *(minutes)*	371 (294 – 434) [n=19]	230 (203 – 322) [n=9]	0.0171
Length of hospital stays *(days)*	10 (7–14) [n=21]	10 (9 – 15) [n=9]	0.5097
Length of NICU/PICU stays *(days)*	3 (2 – 5) [n=19]	3 (2 – 11) [n=9]	0.9717
Re do surgery *(%)*	0 (0%) [n=21]	3 (33%) [n=9]	0.0207
Wall chest anomalies *(%)*	4 (22%) [n=18]	3 (43%) [n=7]	0.3554

Postoperative chest wall anomalies were observed more frequently after thoracotomy than after thoracoscopy (56% vs. 13%), showing a clear trend toward higher incidence that approached but did not reach statistical significance (*p* = 0.0581).

Similarly, children who required redo surgery exhibited a higher rate of chest wall anomalies compared with those who did not undergo additional procedures (50% vs. 26%); however, this difference was not statistically significant (*p* = 0.4900).

## Discussion

The surgical management of CPAM continues to evolve in parallel with advances in prenatal diagnosis and minimally invasive techniques. While the goal of surgery remains the complete removal of the malformed tissue with preservation of healthy parenchyma, the optimal extent of resection (lobectomy versus lung-sparing surgery) remains debated among pediatric surgeons.

Lobectomy has traditionally been regarded as the standard of care for CPAM, offering a definitive solution that eliminates the risk of residual disease within the affected lobe. By removing the entire embryologically abnormal segment of lung tissue, lobectomy minimizes the possibility of postoperative recurrence, infection, or malignant transformation. Several studies have shown that, despite the removal of an entire lobe, long-term pulmonary function is generally preserved owing to compensatory alveolar growth in children. Moreover, lobectomy is associated with reproducible outcomes, low re-intervention rates, and predictable postoperative recovery, making it a safe and effective approach in most cases, as also confirmed in our series.

In our cohort of 31 children with CPAM, 22 (71%) underwent lobectomy and 9 (29%) lung-sparing resections (segmentectomy or wedge). The median operative and anesthesia times were significantly longer for lobectomy compared with lung-sparing procedures, consistent with previous reports that associate lobectomy with a more extensive dissection and careful hilar management. Nevertheless, lobectomy was associated with fewer postoperative complications (19% vs. 44%) and no redo surgeries, whereas redo surgery was required in one-third of patients who underwent lung-sparing resection. Similarly, chest wall anomalies were less frequent following lobectomy (22% vs. 43%), though not statistically significant. These findings align with published series reporting higher rates of recurrence or persistent air leaks after limited resections, especially in children with multilobular or ill-defined lesions.

In contrast, lung-sparing resections aim to preserve functional parenchyma, a particularly attractive goal in small children. These procedures may reduce operative trauma and preserve more alveolar units, potentially offering better long-term ventilatory reserve. However, they are technically demanding and often associated with a higher risk of incomplete excision, prolonged air leak, or residual cystic tissue.

The embryological nature of CPAM adds an additional layer of complexity: the malformation frequently extends microscopically beyond the radiologically visible lesion, involving adjacent portions of the same lobe. Consequently, even an apparently complete segmentectomy may leave behind microscopic areas of dysplastic tissue with a propensity for infection or, albeit rarely, neoplastic transformation.

Experimental evidence further supports this concept. Recent translational data have shown that the regenerative response of CPAM tissue is intrinsically altered when compared with healthy lung parenchyma. In a comparative transcriptomic analysis of mesenchymal stromal cells derived from normal lung tissue and CPAM lesions, Silvestro et al. [8] demonstrated that cells isolated from CPAM exhibit a dysregulated response to hypoxic stimuli, with reduced activation of pathways involved in cytoskeletal organization, epithelial repair, and tissue regeneration. In contrast, healthy lung–derived cells showed a more robust regenerative and metabolic response under the same conditions. These findings suggest that residual CPAM tissue retains an impaired reparative capacity, characterized by downregulation of synthetic, metabolic, and structural pathways essential for effective postnatal lung remodeling. From a surgical perspective, the persistence of microscopic malformative tissue after limited resection may therefore not only predispose to recurrence or infection but also interfere with normal post-resection regenerative processes, ultimately compromising long-term tissue adaptation and functional recovery.

In this light, retaining an affected lobe may not always be the most appropriate strategy for achieving definitive disease control, a notion supported by the 0% redo rate after lobectomy versus 33% after lung-sparing resection in our experience.

With respect to length of hospital stay, no significant difference was observed between patients undergoing lobectomy and those treated with lung-sparing resection. In our series, postoperative hospitalization was comparable between the two groups, despite the longer operative and anesthesia times associated with lobectomy. These findings suggest that the extent of parenchymal resection does not substantially impact length of stay, which appears to be more closely related to postoperative course and complication profile rather than to the type of resection itself.

Finally, the mean age of patients in our cohort was 8 months. According to the current literature, the optimal timing for elective thoracoscopic lobectomy in patients with CPAM has not yet been clearly defined and remains a complex and debated issue [9] While elective resection is commonly performed in asymptomatic children between 6 and 15 months of age, recent reports suggest that procedures performed after 3 months of age may be associated with higher conversion rates compared with those carried out in younger children, often in conjunction with increased bleeding and transfusion requirements. In contrast, surgery performed earlier in life has been associated with shorter operative times, reduced length of hospital stay, and a lower risk of postoperative infectious complications. These findings highlight the need for individualized timing of surgery rather than a rigid age-based threshold.

The advent of adjunctive technologies has improved the precision and safety of minimally invasive approaches. Indocyanine green (ICG)–based fluorescence imaging has been increasingly used to delineate intersegmental planes and define the boundaries between normal and diseased parenchyma. Recent studies in children with CPAM have demonstrated that near-infrared (NIR) fluorescence with intravenous ICG is safe and effective for identifying intersegmental planes during thoracoscopic segmentectomy, with no conversions or recurrences reported [7] Furthermore, atomized or inhalational ICG administration has been proposed to enhance the intraoperative visualization of lesion margins during thoracoscopic resections, enabling more precise parenchymal dissection and reducing the risk of air leaks [7, 10, 11]

Despite these promising advances, fluorescence-guided surgery (FGS) does not change the fundamental embryological pathology of CPAM. When the entire lobe is developmentally abnormal, segmental resection (even when aided by ICG) cannot ensure that microscopic malformations have been completely removed. The theoretical advantage of parenchymal preservation must therefore be weighed against the potential for residual disease and future complications. In our series, although lung-sparing resections were associated with shorter operative times, they also carried a higher risk of incomplete resection, reflected by the need for reoperation and the higher, though not statistically significant, rate of postoperative complications.

Similarly, virtual reality (VR) and three-dimensional (3D) reconstruction technologies are increasingly being integrated into preoperative planning. VR allows surgeons to interactively explore patient-specific anatomy and vascular variants before thoracoscopic surgery, enhancing spatial understanding and operative confidence. These technologies hold great potential for improving surgical training and precision. Yet, while VR may facilitate safer minimally invasive surgery and optimize the use of thoracoscopy, it complements rather than replaces the fundamental principles of complete resection [12, 13]

This study has several limitations that should be acknowledged. First, it represents a retrospective, single-center analysis with a relatively small sample size, which may limit the generalizability of the findings. The surgical procedures were performed by two main operators within the same institution, ensuring technical consistency but potentially introducing operator-dependent bias. Finally, while the study reflects real-world clinical practice, its non-randomized design and limited case selection for lung-sparing procedures may have influenced the observed differences in outcomes.

Despite these limitations, our findings reinforce the evidence that lobectomy remains the safest and most definitive surgical option for CPAM, providing complete removal of the malformation, preventing residual disease, and avoiding the need for re-intervention.

Future directions should focus on prospective, multicenter studies aimed at defining clear selection criteria for lung-sparing resections, standardizing the use of ICG fluorescence and VR planning, and assessing their impact on long-term pulmonary function, recurrence, and quality of life.

Such collaborative efforts will be essential to balance innovation with surgical safety, ensuring that technological progress continues to serve the goal of achieving the best possible outcomes for children with CPAM.

## Conclusion

Lobectomy remains the gold standard surgical treatment for the treatment of CPAM in children. By removing the entire embryologically abnormal lobe, it ensures complete excision, minimizes the risk of residual disease, and provides the most reliable and definitive long-term outcomes. Lung-sparing approaches may represent an appealing alternative in carefully selected patients; however, they currently require further intraoperative innovation and standardization to achieve durable and reproducible results in children with CPAM.

## Data Availability

No datasets were generated or analysed during the current study.
